# Available data do not rule out Ctenophora as the sister group to all other Metazoa

**DOI:** 10.1038/s41467-023-36151-6

**Published:** 2023-02-10

**Authors:** Nathan V. Whelan, Kenneth M. Halanych

**Affiliations:** 1 Southeast Conservation Genetics Lab, Warm Springs Fish Technology Center, US Fish and Wildlife Service, Auburn, AL USA; 2 School of Fisheries, Aquaculture, and Aquatic Sciences, College of Agriculture, Auburn University, Auburn, AL USA; 3 Center for Marine Science, University of North Carolina at Wilmington, Wilmington, NC USA

**Keywords:** Phylogeny, Phylogenetics, Molecular evolution

**arising from** A. K. Redmond & A. McLysaght *Nature Communications* 10.1038/s41467-021-22074-7 (2021)

Redmond and McLysaght (RM)^1^ conclude that the position of Ctenophora as sister to all other animals is unsupported. Here, we contend that this conclusion is not consistent with their analyses. Close inspection of RM’s results and reanalyses indicate that when assessing phylogenetic inference methods using “known” relationships, RM did not discuss all widely-accepted relationships, some of which were unusual and cast doubt on their conclusions about method performance. RM also state that decreased support in some analyses means that the hypothesis of Ctenophora as sister to all other animals is unsupported by the data. However, less than 100% support in some analyses is not zero support, and Ctenophora-sister is the best supported hypothesis in most analyses.

RM examined various approaches to phylogenetic inference on three datasets (BEA, LEAP, LEAN). Accuracy of each approach was based on how well each method recovered benchmark, or widely accepted, relationships. To put these results in fuller context, we reanalyzed RM’s datasets using the 20% relaxed clustering partition testing approach with IQTREE (1.6.12)^2^ and also considered a broader set of widely accepted relationships. Analyses from RM on WEA 17 were not redone, but we more carefully inspected their results on WEA17 using a broader set of widely accepted relationships. RM conclude that only SR4 data recoding (*sensu*^3^) will result in accurate relationships on the LEAP and LEAN dataset. However, the fact that Chordata was recovered as paraphyletic, in contrast to abundant data^4^, is not discussed. Both SR4 analyses on the LEAP dataset and one SR4 analyses on the LEAN dataset resulted in a non-monophyletic Chordata (Fig. 1; Supplementary Figs. 5, 7 in ref. ^1^), indicating inaccuracy of SR4 recoding. Although SR4 recoding results in the accepted relationship of Arthropoda + Platyhelminthes with LEAP and fully supported Nematoda + Arthropoda with LEAN, the failure to recover monophyletic Chordata trades one inaccurate relationship for another. Furthermore, when support values and all possible widely accepted animal relationships^4^ are taken as a whole, non-recoded partitioning with site-heterogeneous models performed better than SR4 recoding on the BEA dataset (Fig. 1). Thus, justifying the use of recoding by citing the method’s inference of select relationships, while not considering its failure to recover other widely accepted relationships, is arbitrary. The inability of any method to recover all accepted relationships on the LEAP and LEAN datasets should call into question the utility of those datasets for assessing method performance.

**Fig. 1:**
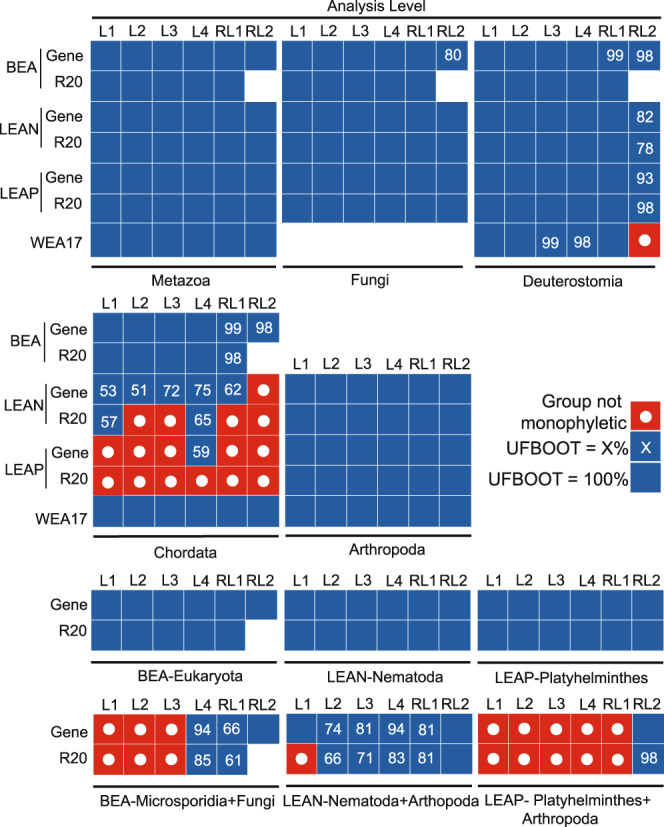
Inference of benchmark relationships following phylogenetic approaches used by Redmond and McLysaght^1^ with partitioning by gene and partitioning using 20% relaxed clustering. Dataset and analysis level abbreviations follow Redmond and McLysaght^1^. “Gene” and “R20” reflect whether the analysis was partitioned by gene or by using 20% relaxed clustering. L1, L2, L3, and L4 are non-recoded analyses with increasing use of site-heterogenous models (see methods of RM). RL1 is recoded analyses without site-heterogeneous models and RL2 is recoded analyses with site heterogeneous models. Solid blue boxes indicate 100% ultrafast bootstrap (UFBOOT) support for the relationship labelled under each set of boxes (e.g., “Metazoa”, “Fungi”). Numbers represent ultrafast bootstrap support less than 100%. Red boxes with dots indicate that a relationship was not recovered. Analysis RL2 for BEA is not reported as IQTREE 1.6.12 tree inference failed because of an error, likely resulting from overparameterization.

In dismissing past work indicating problems with amino acid recoding^5^, RM claim that failure to recover some accepted relationships is expected with SR4 recoding. They state that other lines of evidence can be used to indicate which relationships inferred with SR recoding are the accurate relationships. RM then use that statement to claim that the Porifera-sister hypothesis is accurate. These claims are logically questionable for two reasons: 1) the first claim ignores poor methodological performance if at least one preferred relationship is recovered, and 2) most analyses have greater support for ctenophores sister at the partition-specific support level (Fig. 4 in ref. ^1^), so even if claim 1 was accepted, RM lack other lines of evidence to support the assertion that SR4 recoding is accurately inferring sponges as the sister group to all other animals. Moreover, the inability of SR4 recoding with site-heterogeneous models to recover a monophyletic Chordata on LEAP and LEAN or monophyletic deuterostomes on WEA17 indicates problems with SR4 recoding. Accepting SR4 recoding as accurate requires one to overlook some parts of the tree while focusing only on particular relationships of interest. Ideally, a robust method should recover all relationships known to be well-supported, which is true of non-recoded analyses on the WEA17 dataset. A simpler explanation that does not require ignoring certain parts of the tree is that SR recoding is inaccurate because it removes information content, rather than reducing systematic error^6^.

All non-recoded analyses done in RM recover Ctenophora as the sister lineage of all other animals. Yet, RM claim that Ctenophora-sister is implausible. A primary line of evidence for this conclusion is decreased support values for Ctenophora-sister with more complex models. However, Ctenophora-sister is the best supported hypothesis in almost every analysis, including those that use the most complex models. Contrary to RM’s claims, decreased support for Ctenophora-sister in some analyses does not indicate unequivocal support for sponges-sister. Taxon sampling is also widely accepted as aiding accurate phylogenetic inference^7^. Notably, all non-recoded analyses with the most taxon-rich dataset, WEA17, strongly support Ctenophora-sister. Phylogenetic inference with SR4 recoding and site-heterogeneous models was the only analysis on WEA17 that did not recover Ctenophora-sister, but it failed to recover deuterostome monophyly (Supplementary Fig. 25 in ref. ^1^). RM also indicated problems with overparameterization in their analysis of WEA17 with SR4 and site-heterogeneous models. We agree, and a recent study indicated that overparameterization can drive inference of sponges-sister^6^. Thus, the most reliable analyses of RM indicate Ctenophora-sister as the most likely hypothesis.

Careful reading indicates that numerous criticisms made by RM about other studies are unwarranted. For example, SR recoding failed to recover accepted relationships on most datasets (Fig. 1; Supplementary Information of ref. ^1^), indicating that concerns raised by Hernandez and Ryan^5^ about data recoding were inappropriately dismissed. Hernandez and Ryan^5^ robustly tested amino acid recoding in simulation, whereas most other studies have relied on assumed relationships (e.g., Porifera sister to all other metazoans; see^6^). Given that empirical phylogenies cannot be known with absolute certainty, simulation approaches are strong evidence that recoding is problematic. Thus, dismissing Hernandez and Ryan^5^ and preferring results with SR4 recoding, even when recoding caused non-monophyly of accepted clades, is problematic.

RM also state that the REA, WEA15, and WEA17 datasets are filled with paralogs that negatively influence phylogenetic inference without providing evidence aside from unpublished “personal communication.” Given the nature and gravity of this debate, unpublished “personal communication” is unreliable evidence as it cannot be easily verified. Importantly, WEA15 and WEA17 were curated to control for paralogs using tree-based approaches^8^. Although we disagree with the premise that WEA15 and WEA17 are fundamentally flawed, if the datasets are as problematic as claimed, then they are unsuitable for inferring relationships, and RM’s conclusions would need to be rejected in favor of acknowledged uncertainty in the phylogenetic position of sponges and ctenophores. Finally, the criticisms of RM about Whelan and Halanych^9^, a study that compared the CAT models of PhyloBayes^10^ to partitioning, are without basis as RM did not perform analyses with the CAT models of PhyloBayes.

Although we support attempts to better model substitutional heterogeneity, methods must be robustly tested. We agree with RM and others that combining partitioning and site-heterogeneous models in a maximum likelihood framework may be a computational tractable and accurate approach for phylogenomic inference^11^ (Fig. 1). Notably, non-recoded partitioning with linked branches and site-heterogeneous models recovered the Ctenophora-sister hypothesis^1^ (Fig. 1). When testing approaches, an objective lens must be applied to assess support and rejection of alternative hypotheses. RM’s presentation does not accurately reflect the level of support in their analyses for the Ctenophora-sister hypothesis. Moreover, the Porifera-sister hypothesis is not viewed with the same critical lens. Researchers are actively generating data from more taxa and genes, which will hopefully shed light on this challenging phylogenetic issue.

## Methods

Maximum likelihood trees were inferred with non-recoded and SR^3^ recoded datasets BEA, LEAP, and LEAN from RM^1^. Following RM^1^, six analyses were done on each dataset (i.e., L1, L2, L3, L4, RL1, RL2) to test for the influence of including site-heterogenous models in analyses. Best-fit partitions and substitution models were inferred with ModelFinder^12^, as implemented in IQ-TREE 1.6.12^2^, with 20% relaxed clustering; branch lengths were linked and each partition was allowed to have its own evolutionary rate. For model testing, L1 analyses included only standard site homogeneous protein subtitution models (e.g., Dayhoff, JTT), L2 analyses included all models from L1 analyses and multi-matrix models (e.g., EX2, LG4M), L3 analyses included all models from L1 and L2 analyses plus multi-profile models with Poisson exchangeabilities (e.g., C10, C20), and L4 analyses included all models from L1, L2, and L3 analyses plus multi-profile models with non-Poisson exchangeabilities (e.g., JTT + C10, LG-C30). Model testing for RL1 analyses included site-homogeneous nucleotide (i.e., 4-state) models. Model testing for RL2 analyses included all models from RL1 analyses plus multi-profile site-heterogenous models with either Poisson or GTR exchangeabilities. All analyses included testing models with a parameter for rate heterogeneity. Following RM^1^, partition finding was done only on L1 analyses; model-testing for L2, L3, L4, RL1, and RL2 analyses was done with the best-fit partitions from L1 analyses on each dataset. Model testing, as described above, was consistent with what was done by RM on the same datasets but with IQ-TREE 1.6.12. Tree inference was done with IQ-TREE using best-fit paritions and models. Support was assessed with 1000 ultrafast boostrap replicates^13^.

### Reporting summary

Further information on research design is available in the Nature Portfolio Reporting Summary linked to this article.

## Supplementary information

Description of Additional Supplementary Files

Supplementary Data 1

Reporting Summary

## Data Availability

Raw data were downloaded from the FigShare reposiroty of ref. ^1^ (10.6084/m9.figshare.12746972.v1). Tree files are available in Supplementary Data 1.
