# Supplementary material for: Available data do not rule out Ctenophora as the sister group to all other Metazoa
Source: Nat Commun. 2023 Feb 10;14:711. doi: 10.1038/s41467-023-36151-6 (PMC9918479; doi:10.1038/s41467-023-36151-6)
Supplement: Supplementary file 1 — Description of Additional Supplementary Files [file 41467_2023_36151_MOESM1_ESM.pdf]

Description of Additional Supplementary Files

File Name: Supplementary Data 1
Description: Zip file of tree files in Newick Format.
